# Significance of Adipose Tissue Maintenance in Patients Undergoing Hemodialysis

**DOI:** 10.3390/nu13061895

**Published:** 2021-05-31

**Authors:** Senji Okuno

**Affiliations:** Kidney Center, Shirasagi Hospital, 7-11-23, Kumata, Higashisumiyoshi-ku, Osaka 546-0002, Japan; okuno@shirasagi-hp.or.jp; Tel.: +81-6-6714-1661

**Keywords:** fat mass, visceral fat, subcutaneous fat, nutrition, mortality, body mass index, obesity paradox

## Abstract

In the general population, obesity is known to be associated with adverse outcomes, including mortality. In contrast, high body mass index (BMI) may provide a survival advantage for hemodialysis patients, which is known as the obesity paradox. Although BMI is the most commonly used measure for the assessment of obesity, it does not distinguish between fat and lean mass. Fat mass is considered to serve as an energy reserve against a catabolic condition, while the capacity to survive starvation is also thought to be dependent on its amount. Thus, fat mass is used as a nutritional marker. For example, improvement of nutritional status by nutritional intervention or initiation of hemodialysis is associated with an increase in fat mass. Several studies have shown that higher levels of fat mass were associated with better survival in hemodialysis patients. Based on body distribution, fat mass is classified into subcutaneous and visceral fat. Visceral fat is metabolically more active and associated with metabolic abnormalities and inflammation, and it is thus considered to be a risk factor for cardiovascular disease and mortality. On the other hand, subcutaneous fat has not been consistently linked to adverse phenomena and may reflect nutritional status as a type of energy storage. Visceral and subcutaneous adipose tissues have different metabolic and inflammatory characteristics and may have opposing influences on various outcomes, including mortality. Results showing an association between increased subcutaneous fat and better survival, along with other conditions, such as cancer or cirrhosis, in hemodialysis patients have been reported. This evidence suggests that fat mass distribution (i.e., visceral fat and subcutaneous fat) plays a more important role for these beneficial effects in hemodialysis patients.

## 1. Introduction

In patients with chronic kidney disease (CKD), especially those undergoing maintenance hemodialysis therapy, poor nutritional status is a common and important complication. Various terms and definitions have been applied for conditions associated with malnutrition, such as loss of muscle and fat tissue, and inflammation, including uremic malnutrition [1], protein–energy malnutrition [2], malnutrition–inflammation atherosclerosis syndrome [3], and malnutrition–inflammation complex syndrome [4]. To avoid confusion, the International Society of Renal Nutrition and Metabolism (ISRNM) has recommended the term protein–energy wasting (PEW) [5]. The four main established categories now recognized for diagnosis of PEW are biochemical criteria and include low body weight and reduced total body fat, weight loss, decrease in muscle mass, and low protein or energy intake.

Several studies have shown that PEW is a significant risk factor for low quality of life, muscle weakness, hospitalization, and mortality; thus, nutritional assessment of hemodialysis patients is important to avoid development and progression of PEW. Body fat mass is considered to be a nutritional parameter, since low fat mass is one of the factors examined for diagnosis of PEW. In the general population, an increase in fat mass, particularly visceral fat mass, is considered to be a risk factor for cardiovascular disease (CVD) and mortality, whereas in hemodialysis patients, such an increase may represent improved nutritional status and better survival. This review focuses on the associations of fat mass and its distribution (i.e., visceral fat and subcutaneous fat) with outcomes, particularly mortality, in patients undergoing hemodialysis. The purpose of this article is to clarify an ambiguous role of fat mass, so-called reverse epidemiology, with the difference among the general population and hemodialysis patients. The search terms I used to collect the bibliography were fat mass, adipose tissue, obesity, dialysis, kidney, mortality, and nutrition. A comprehensive literature search was preformed using PubMed from January 1980 to January 2021.

## 2. Higher Body Mass Index Is Related to Better Survival in Hemodialysis Patients

Obesity, primarily defined by excess body fat mass, is highly prevalent and increasing worldwide and has been found to be associated with serious outcomes, including diabetes, hypertension, CVD, and mortality [6,7]. Body mass index (BMI) is considered to be a reasonably good measure of general adiposity, and thus, the most common method for defining obesity is based on that. Despite the high risk of adverse outcomes in the general population, high BMI may be associated with a survival advantage in CKD patients, which is known as the obesity paradox or reverse epidemiology of obesity [8,9]. Other than CKD, the obesity paradox has also been observed in various clinical settings, including cases of chronic heart failure [10], chronic obstructive pulmonary disease [11], and cancer [12], as well as elderly patients [13].

In 1982, Degoulet et al. reported that high BMI in 1453 French hemodialysis patients was not associated with cardiovascular mortality or all-cause mortality [14]. Using data from the United States Renal Data System (USRDS), Leavey et al. also found no evidence of increased mortality risk related to higher values of BMI in 3607 hemodialysis patients (BMI 24.4 ± 5.3 kg/m^2^ (mean ± SE)) in follow-up examinations conducted over a five-year period [15]. In another study, Fleischmann et al. noted that compared with normal-weight (BMI 20–27.5 kg/m^2^), the one-year survival rate was significantly greater in overweight hemodialysis patients (BMI ≥ 27.5 kg/m^2^, 38% of 1346 patients), a paradoxical finding when compared to the general population [16]. Additionally, that study showed that with a one-unit increase in BMI over 27.5 kg/m^2^, the relative risk for mortality was reduced by 30% (*p* < 0.04).

Johansen et al. investigated the relationship between BMI and survival in USRDS data obtained for 418,055 patients who had started hemodialysis [17]. They found that high BMI was associated with increased survival over a two-year averaged follow-up period after adjustments for demographic, laboratory, and comorbidity data, even in subjects with extremely high BMI. Furthermore, high BMI was also associated with reduced risk of hospitalization. These results were observed for Caucasian, African American, and Hispanic subjects, but not for Asians. Additionally, alternative estimates of adiposity, including the Benn index and estimated fat mass, yielded similar results, and adjustments for lean body mass did not substantially alter the findings. A meta-analysis was also conducted, which showed that for every 1 kg/m^2^ increase in BMI, the reduction in risk of all-cause mortality was 3% (hazard ratio (HR) 0.97; 95% CI 0.96–0.98, *n* = 89,332), and the risk of cardiovascular mortality was reduced by 4% (HR 0.96; 95% CI 0.92–1.00, *n* = 8918) in the study cohort [18].

Hemodialysis patients can often experience fluctuations in body weight resulting from changes in dietary intake and comorbidity status, and several studies have shown that body weight changes are strongly associated with mortality [19]. Chazot et al. conducted a prospective observational study of 5592 incident hemodialysis patients (age 64.4 ± 16.5 years, 40.9% females, 27.7% diabetics) in Southern Europe who were followed for 2.0 ± 1.6 years [20]. The four categories used for baseline BMI—underweight, normal range, overweight, and obese—were found to significantly influence survival. With the normal BMI range used as a reference, HR (95% CI) was 0.74 (0.67–0.90) for overweight and 0.78 (0.56–0.87) for obese patients, suggesting an association of higher BMI, even in obese subjects, with better survival. Moreover, when compared to patients in whom body weight remained stable during the first year after initiation of hemodialysis, survival was significantly lower in those with a decrease in body weight (less than −5.8% in one year) with an HR of 1.60 (95% CI 1.20–2.14).

Kalantar-Zadeh et al. explored the effects of both baseline and changes in BMI on all-cause and cardiovascular mortality in a two-year study of 54,535 patients receiving maintenance hemodialysis in the United States (age 61.7 ± 15.5 years, 54% males, 45% diabetics) [21]. They found that obesity, including morbid obesity, was associated with better survival and reduced cardiovascular death even after accounting for changes in BMI and laboratory values over time. In examinations of the regression slope of change in weight over time, progressively worsening loss was associated with poor survival, whereas weight gain showed a tendency for decreased cardiovascular death.

Chang et al. examined the association of changes in body weight after initiation of hemodialysis treatment with all-cause mortality in a study conducted in the United States that included 58,106 patients [22]. Compared with the reference group (−2% to 2% weight change occurring between first and fifth months), the mortality HR (95% CI) value during the first five months for patients with −6% to −2% weight loss was 1.08 (1.02–1.14), while that in those with greater than or equal to −6% weight loss was 1.14 (1.07–1.22). When weight changes from 5 to 12 months were considered, the association between such change and mortality was even stronger. Each 4% increase in weight between the 5th and 12th months was associated with an HR value of 0.92 (95% CI 0.90–0.93; *p* < 0.001), whereas the same degree of weight change was associated with an HR value of 0.96 (95% CI 0.95–0.98; *p* < 0.001) over the first 5 months. 

Doshi et al. examined the association between BMI and all-cause mortality in a study of 123,624 adult maintenance hemodialysis patients in the United States (age 61 ± 15 years, 45% females, 32% African Americans) using marginal structural model analysis, a technique that accounts for time-varying confounders [23]. They confirmed that BMI had a linear incremental inverse association with mortality. In marginal structural model analysis results, as compared with the reference (BMI 25 to <27.5 kg/m^2^), BMI < 18 kg/m^2^ was associated with 3.2-fold higher death risk (HR 3.17; 95% CI 3.05–3.29), while mortality risk declined with increasing BMI, with the greatest survival advantage, 31% lower risk (HR 0.69; 95% CI 0.64–0.75), observed in patients with a BMI value ranging from 40 to <45 kg/m^2^. 

Stenvinkel et al. examined the relationship between BMI and all-cause mortality in 5904 European incident hemodialysis patients, accounting for inflammation [24]. Patients were classified based on the presence (*n* = 3231) or absence (*n* = 2673) of inflammation (defined as C-reactive protein ≥10 mg/L and /or albumin ≤35 g/L) and then further divided into quintiles by BMI. Higher BMI was associated with lower mortality risk in patients with inflammation, whereas no protective effect was associated with the higher BMI quintile in patients without inflammation. In the Dialysis Outcomes and Practice Patterns Study (DOPPS), Leavey et al. evaluated relationships between BMI and mortality in hemodialysis subpopulations defined by continent, ethnicity, gender, tertile of illness severity (based on a score derived from comorbid conditions and serum albumin concentration), age, smoking, and diabetic status [25]. The relative mortality risk was found to decrease with increasing BMI. That result was statistically significant in all subjects, except for the smallest subgroup of patients who were <45 years old and also in the healthiest tertile of comorbidity. 

It has also been reported that the association between weight change and mortality is less apparent in obese hemodialysis patients. In 6296 European patients with prospective data collected every 6 months for 3 years (age 64 ± 14 years, 61% males, 31% diabetics, BMI: 25.3 ± 4.9 kg/m^2^), Cabezas-Rodriguez et al. examined the influence of BMI on the association of short-term weight change with mortality [26]. Compared with stable weight (±1%), weight loss (>1% weight decline) in the whole cohort was strongly associated with higher mortality, while weight gain (>1% weight increase) had an association with lower mortality. After stratification by BMI categories, this remained true when nonobese categories were used, especially in underweight patients. As for obese patients, the association of weight loss with mortality was attenuated and no longer statistically significant (HR 1.28; 95% CI 0.74–2.14), and no survival benefit was seen in association with gaining weight (HR 0.98; 95% CI 0.59–1.62), indicating potential resistance to the development of wasting in obese hemodialysis patients. These results suggested that the association between body size and mortality in hemodialysis patients might be affected by baseline health and nutritional status. 

Previous studies of the association between BMI and mortality have shown conflicting results obtained with nondialysis-dependent CKD patients [27]. Madero et al. reported that high BMI had no protective effect on 1759 CKD patients with a mean estimated glomerular filtration rate (eGFR) of 39 ± 21 mL/min/1.73 m^2^ [28]. In 920 Swedish patients with advanced kidney dysfunction (serum creatinine level >3.4 mg/dL in males, >2.8 mg/dL in females), Evans et al. reported that high BMI was associated with lower mortality [29]. Similarly, in a study of 521 male veterans in the United States with CKD (age 68.8 ± 10.4 years, eGFR 37.5 ± 16.8 mL/min/1.73 m^2^), Kovesdy et al. reported that higher BMI was associated with lower mortality in groups with BMI in the 10th to 50th, 50th to 90th, and >90th percentiles versus the <10th percentile, noting values of 0.75 (95% CI 0.46–1.22), 0.56 (95% CI 0.33–0.94), and 0.39 (95% CI 0.17–0.87), respectively [30]. Lu et al. examined the associations of BMI with all-course mortality and disease progression in a cohort of 453,946 United States veterans with nondialysis-dependent CKD (eGFR < 60 mL/min/1.73 m^2^) [31]. Their results showed a relatively consistent U-shaped association of BMI with clinical outcomes. For example, BMI ≥ 35 mg/m^2^ was associated with worse outcome in patients with an earlier stage of CKD, while that association was attenuated in those with eGFR < 30 mL/min/1.73 m^2^. 

## 3. Determination of Visceral and Subcutaneous Fat and Muscle Mass

BMI is the most commonly used measure for the assessment of obesity in both research and clinical practice, though it does not distinguish between fat and lean mass [32,33]. Thus, even though greater BMI has beneficial effects for survival, fat and lean mass should be separately evaluated to elucidate the association of body composition with mortality. 

Anthropometry is widely used for determining body composition because of its ease to use, wide availability, low cost, and favorable safety profile, though fluid status has been shown to influence calculations in hemodialysis patients [34,35]. Mid-arm circumference (MAC) and mid-arm muscle circumference (MAMC) are anthropometric methods developed to assess lean body mass, while skinfold thickness (SFT) at four sites (triceps, biceps, subscapular, ileac crest) is used to assess total body fat mass. For assessing abdominal obesity, waist circumference, waist-to-hip ratio, and waist-to-height ratio are applied. Kamimura et al. evaluated body fat mass using skinfold thickness and a bioelectrical impedance analysis (BIA) technique in 90 clinically stable hemodialysis patients. Body fat mass measurements based on skinfold thickness (13.5 ± 6.2 kg) and BIA (13.7 ± 6.7 kg) were similar, and strong correlations were found between results obtained with these two methods (r = 0.87) [36]. Oe et al. also noted that values for lean mass and fat mass obtained via skinfold anthropometry and BIA were significantly correlated [37].

BIA, a noninvasive rapid and reliable method with low cost, is also commonly used to evaluate body composition for both epidemiological and clinical purposes [38]. A principle of BIA techniques is that the transit time of a low-voltage electric current through the body is dependent on body composition characteristics [39].

Dual energy X-ray absorptiometry (DXA) is considered to be a more accurate and reliable reference method for measurements of fat and lean mass. With this method, the differential of two X-ray beams as they pass through the body is determined so as to distinguish bone from soft tissue, then the findings are subsequently used to divide soft tissue mass into fat and fat-free soft-tissue mass (lean mass). However, measurements of lean mass by DXA are influenced by hydration, whereas that has scarce effects on fat mass measurements [40,41]. Reproducibility of fat mass measurements by DXA has also been shown to be excellent, with variations <2% among patients undergoing hemodialysis [42]. Nishizawa et al. showed that DXA-determined fat mass index (kg/m^2^) in 104 patients undergoing hemodialysis (age 53.9 ± 9.1 years, hemodialysis duration 7.5 ± 5.1 years, 39 males and 64 females) was 5.2 ± 0.2 kg/m^2^, significantly lower than the value obtained with 167 age- and gender-matched healthy control subjects of 5.8 ± 0.2 kg/m^2^ (age 52.9 ± 9.0 years, 53 males and 114 females) (*p* < 0.05) [42].

The most reliable methods for determining body composition in clinical practice might be computed tomography (CT) and magnetic resonance imaging (MRI) [35], as both can be utilized to assess fat mass distribution and also distinguish between subcutaneous and visceral fat with direct measurements. The results obtained are accurate and reproducible as compared with simulated phantom studies. However, CT and MRI are impractical for general population screening due to their high cost, and CT exposes the subject to radiation. Although DXA cannot directly discriminate between visceral and subcutaneous fat, visceral fat mass estimated by DXA has been shown to be strongly correlated with the visceral fat area determined by CT and visceral fat mass by MRI [43].

## 4. Fat Mass—A Useful Nutritional Marker and Its Significance for Survival

It is considered that fat mass may serve as an energy reserve against a catabolic condition, and the capacity to survive starvation has also been shown to be dependent on its amount [44]. In obese model rats developed by a high-fat diet, increased fat mass provided fuel availability and conservation of lean mass with starvation compared to control rats [45]. Therefore, fat mass is regarded as an important indicator of nutritional status in hemodialysis patients. Additionally, low fat mass, one of the characteristics examined for PEW diagnosis [5], is often complicated in hemodialysis patients and thought to contribute to the high rates of morbidity and mortality observed in those cases. Consequently, it is assumed that fat mass is decreased in hemodialysis patients with PEW. In a study of 468 prevalent hemodialysis patients (median age 66 years, median hemodialysis duration 37 months, 34% females, 50% diabetics), Anton-Perez et al. reported the results of a multivariate regression analysis showing a linear inverse relationship between lower fat mass determined by BIA with a greater number of PEW syndrome categories [46]. Similarly, in 186 advanced CKD patients (age 66.1 ± 16 years, 101 males and 85 females), Perez-Torres et al. found that the prevalence of PEW was 30.1% and evidence of lower fat mass in PEW patients [47].

Various clinical and biochemical parameters can be used to evaluate nutritional status in individuals with CKD. Among nutritional scoring systems, subjective global assessment (SGA) and malnutrition–inflammation score (MIS) are often utilized. SGA is based on a combination of subjective and objective features obtained from patient medical history and physical examination findings. In a comparison of SGA with nutritional status determined by total-body nitrogen, which directly quantifies body protein content, SGA was shown to be able to differentiate severely malnourished dialysis patients from those with normal nutrition [48]. A modified version of SGA was used in the DOPPS, which found that lower values obtained with that modified version were associated with higher risk of mortality in hemodialysis patients [49]. Kalantar-Zadeh et al. examined 41 hemodialysis patients (age 57 ± 12 years, hemodialysis duration 3.0 ± 2.1 years, males 49%) using the modified SGA system and showed that subcutaneous fat mass assessed by biceps skinfold thickness was significantly negatively correlated with malnutrition score [50]. In another study, Paudel et al. found that 96 (21%) of 455 peritoneal dialysis patients were malnourished, based on an SGA score between 1 and 5, and those malnourished patients exhibited a significantly lower fat tissue index determined by BIA [51]. In a study of 1334 older adults (age ≥ 65 years) not receiving dialysis with advanced CKD and an eGFR <20 mL/min/1.73 m^2^, Windahl et al. reported that fat mass was decreased according to the SGA subscale [52]. 

MIS is an adaptation of SGA for hemodialysis patients and has 10 components derived from medical history, physical examination findings, BMI, and laboratory parameters such as serum albumin and transferrin level [53]. Studies have shown that MIS results can be used to predict poor outcome in hemodialysis, peritoneal dialysis, and nondialyzed CKD patients [54,55]. Wang et al. investigated 144 CKD stage 1–4 patients (median age 53 years; IQR 38–63 years, 94 males and 50 females) and reported that MIS was negatively correlated with BIA-measured fat tissue index (r = −0.179, *p* = 0.032) [56]. In another study that included 91 patients (age 60 ± 14 years, 70.3% males, BMI 24 ± 4.1 kg/m^2^) being treated with hemodialysis, Arias-Guillen et al. compared body composition evaluated by BIA between groups with or without PEW with that defined as MIS above 5 and reported that subjects with PEW showed a significantly lower fat tissue index [57].

## 5. Factors Affecting Fat Mass Changes

### 5.1. Nutritional Support

Several studies have shown that nutritional interventions, such as oral nutritional supplementation and intradialytic parenteral nutrition, can improve the nutritional status of hemodialysis patients [58]. Pupim et al. reported that protein turnover study results showed a highly positive whole body net balance during hemodialysis for both intradialytic parenteral nutrition and oral supplementation as compared with a control group [59]. In addition, skeletal muscle protein homeostasis examined during the hemodialysis session was also found to be improved with both intradialytic parenteral nutrition and oral supplementation. Furthermore, Fouque et al. found that use of energy-dense phosphate-restricted renal-specific oral supplementation in hemodialysis patients with low protein intake resulted in improved SGA and quality of life [60]. 

Improvement of nutritional status with oral supplementation is also indicated by an increase in fat mass, with fat mass change considered to function as a parameter indicating nutritional changes. Caetano et al. investigated the effects of intradialytic oral supplementation along with a protein-rich meal on body composition in 99 hemodialysis patients with a serum albumin level <38 g/L (age 69.9 ± 12.9 years, hemodialysis duration 60.0 ± 50.0 months) [61]. In the intervention group, patients ate a protein-rich meal during each treatment session for six months, while the control group ingested their usual snack brought from home. Although lean mass was decreased in both the intervention and control groups, fat mass at the end of the study was significantly increased in the intervention group in contrast to a decline in the control. Similarly, in 36 patients undergoing hemodialysis, Martin-Alemany et al. found that fat mass assessed by triceps skinfold thickness was significantly increased in association with oral nutritional supplementation or that and resistance exercise for a period of 12 weeks [62]. 

Nutritional supplementation may also improve survival of hemodialysis patients. Weiner et al. reported that oral protein supplementation given during the dialysis procedure was associated with a 29% reduction in risk of all-cause mortality (HR 0.71; 95% CI 0.58–0.86) in hemodialysis patients with a serum albumin level ≤3.5 g/dL [63]. Similarly, Lacson et al. found mortality risk decreased to 0.91 (95% CI 0.85–0.98) in intention-to-treat analysis and 0.66 (95% CI 0.61–0.71) in as-treated analysis, after adjustment for confounding factors, in hemodialysis patients with serum albumin ≤3.5 g/dL [64]. These results suggest that fat mass increased by nutritional supplementation in hemodialysis patients might be associated with survival improvement. 

### 5.2. Initiation of Hemodialysis

Following initiation of hemodialysis, most patients generally gain a sense of improved wellbeing and better appetite, as most symptoms associated with uremia, such as loss of appetite, nausea, and general fatigue, are diminished or disappear. Thus, it is considered that nutritional status is improved by initiation of hemodialysis, and in an investigation of a stable cohort, Goldwasser et al. reported that serum albumin and creatinine levels rose by 12% to 13% during the first half-year of hemodialysis [65]. Fat mass as a nutritional marker may also increase following initiation of hemodialysis because of improved nutritional status. We conducted a study of changes in fat mass in hemodialysis patients and found that it was increased in the first year after initiation of treatment [66]. In 72 patients with CKD (age 62 ± 12 years, 42 males and 30 females), body fat mass was determined by DXA at one month after initiation of maintenance hemodialysis and again approximately one year later. The second measurement showed significantly greater fat (11.38 ± 3.84 vs. 10.09 ± 4.12 kg, *p* < 0.0001). Additionally, change in fat mass per month was negatively correlated with baseline serum albumin concentration (r = −0.449; *p* < 0.0001) and basal fat mass (r = −0.423; *p* < 0.001). Our results suggested that fat mass increase after initiation of hemodialysis may be greater in patients with worse initial nutritional status. In a study of 8227 incident hemodialysis patients, Marcelli et al. also found that fat mass index evaluated by BIA was increased by approximately 0.95 kg/m^2^ in the first 2 years after the start of hemodialysis [67]. Similar to our results, in addition to female gender and diabetes status, basal fat mass index was found to be associated with a significantly greater increase in fat mass index. Similarly, Keane et al. observed a mean increase in fat mass of 0.65 kg determined by BIA over a 2-year follow-up period after initiation of hemodialysis in 299 patients [68]. 

In a cross-sectional study, we examined the association between fat mass and hemodialysis duration to clarify the period of increasing fat mass [69]. In 561 patients with a hemodialysis duration less than 180 months (age 62.3 ± 11.5 years, 336 males and 225 females), fat mass index for each year of hemodialysis tended to increase during the first three years of treatment and then showed a decreasing trend thereafter. The fat mass index value for the third year (5.85 ± 2.92 kg/m^2^) was significantly higher compared to the other years. Furthermore, that index was positively correlated with hemodialysis duration in the first three years (r = 0.124; *p* < 0.05), and then a negative correlation was seen with duration greater than three years (r = −0.192; *p* < 0.001). These results indicated increases in fat mass during the first three years after hemodialysis initiation.

### 5.3. Inflammation

Inflammation is well known to be strongly associated with malnutrition in CKD patients, and a decrease in fat mass can be predicted in those in a state of high inflammation. We conducted a study to examine the association of inflammation, represented by C-reactive protein (CRP) level, with changes in fat mass in 389 hemodialysis patients who had a treatment duration of more than one year [70]. Body fat mass was determined twice using DXA, with a one-year interval between measurements. The results showed that change in fat mass was significantly negatively correlated with CRP (r = −0.165; *p* < 0.005). Additionally, multiple regression analysis indicated that CRP significantly (β = −0.163; *p* < 0.005) affected fat mass change, independent of hemodialysis duration, serum albumin level, baseline fat mass, and other confounding clinical factors (R^2^ = 0.127; *p* < 0.001). 

### 5.4. Diabetes

Several studies have shown that the presence of diabetes is associated with poor nutritional status in CKD patients [71]. We compared changes in fat mass in hemodialysis patients with and without diabetes by determining body fat mass twice by DXA with a 1-year interval in 217 male hemodialysis patients (age 60 ± 13 years, 32% diabetics) who had a duration of hemodialysis from 1 to 10 years (4.9 ± 2.5 years) [72]. Body fat mass was significantly decreased from 12.1 ± 4.4 to 11.0 ± 4.7 kg (*p* < 0.01) during the 1-year study period in the diabetes patients, whereas that was not seen in the nondiabetes patients (12.2 ± 5.0 vs. 11.9 ± 4.9 kg; *p* = 0.15). Further, percentage change in fat mass in one year in the diabetes patients was significantly greater than that in those without diabetes (−7.9 ± 3.4% vs. 0.1 ± 1.9%; *p* < 0.05). Of several clinical parameters examined, protein catabolic rate had a significantly positive correlation with change in fat mass, suggesting that poor protein intake may be a risk factor for fat mass decrease. 

## 6. Fat Mass and Mortality

In the general population, it is considered that the relationships of body fat mass and BMI with mortality are similar. The association between body fat mass (assessed by BIA) and all-cause mortality was investigated by Bigaaed et al. in 27,178 males and 29,875 females in the general population (age 50–64 years) [73]. The median follow-up period was 5.8 years, and 1851 died during the study. They found a J-shape association of body fat mass index with mortality in both the male and female subjects.

Although excess body fat mass is a risk factor for mortality in the general population, high fat mass in hemodialysis patients appears to be protective. In a study of 808 Japanese hemodialysis patients (age 55.1 ± 11.4 years, hemodialysis duration 70.1 ± 66.2 months, 61.3% males, 19.9% diabetics), Kakiya et al. reported that increased fat mass index determined by DXA was associated with decreased all-cause mortality (HR 0.926; 95% CI 0.891–0.962; 1 kg/m^2^ increase) and noncardiovascular mortality (HR 0.850; 95% CI 0.806–0.896; 1 kg/m^2^ increase) during the mean follow-up period of 53 months, after adjustment for confounding variables such as diabetes, serum albumin, and creatinine level [74]. In addition, higher lean mass index was associated with lower cardiovascular mortality (HR 0.874; 95% CI 0.814–0.938; 1 kg/m^2^ increase). Similarly, Honda et al., in an investigation of 328 CKD patients starting dialysis (age 53 ± 12 years, 201 males), found that low fat mass index (evaluated by DXA) was an independent predictor of higher mortality during a six-year follow-up period, after adjustments for age, diabetes, CVD status, inflammation, and other confounders (HR 2.179; 95% CI 1.058–4.488; *p* = 0.0345; low tertile as compared with others) [75]. 

Yajima et al. analyzed the association of fat mass (determined by BIA) and mortality in 162 hemodialysis patients. During the follow-up period (median 2.5 years, range 1.0–4.5 years), 29 of the subjects died. Univariate Cox proportional hazards analysis showed that both higher BMI (HR 0.87; 95% CI 0.76–0.98; *p* = 0.022) and fat tissue index (HR 0.86; 95% CI 0.74–0.98; *p* = 0.021) were significant predictors of lower all-cause mortality. In findings obtained with multivariate Cox proportional hazards analysis after adjusting for age, gender, albumin, diabetes, hypertension, and history of CVD, the adjusted HR value was 0.40 (95% CI 0.17–0.90; *p* = 0.027), above the median value for the higher fat tissue index group [76].

In another study, Noori et al. analyzed 742 maintenance hemodialysis patients (age 54 ± 15 years, dialysis duration 28 ± 26 months, 53% diabetics, BMI 26.5 ± 5.8 kg/m^2^, 31% African Americans), with males (*n* = 391) and females (*n* = 351) categorized separately into four quartiles based on near-infrared interactance-determined fat and lean mass. After adjustments for case-mix and inflammatory markers, the highest quartiles for fat mass and lean mass in females were both associated with greater survival compared to the lowest quartile, with estimated HR values of 0.38 (95% CI 0.20–0.71) and 0.34 (95% CI 0.17–0.67), respectively. In males, the highest quartile for fat mass but not lean mass was associated with greater survival, and those had estimated HR values of 0.51 (95% CI 0.27–0.96) and 1.17 (95% CI 0.60–2.27), respectively [77]. Marcelli et al. investigated an international European cohort of 37,345 hemodialysis patients (age 62.7 ± 15.2 years) and found that low fat tissue index (<10th percentile), determined with BIA, was associated with significantly increased mortality compared to the reference fat tissue index between the 10th and 90th percentile (HR 1.19; 95% CI 1.08–1.31; *p* < 0.001) [78]. Moreover, Caetano et al. [79] and Duong et al. [80] reported that low fat mass determined using BIA was significantly associated with higher risk of mortality in hemodialysis patients. Based on these results, it is suggested that greater fat mass is associated with better survival in hemodialysis patients in contrast to the general population. 

We performed a study to examine the influence of fat mass change on mortality during a 1-year interval in 190 female patients (age 61.9 ± 11.3 years, hemodialysis duration 7.2 ± 6.4 years, 26.3% diabetics, BMI 20.3 ± 3.2 kg/m^2^, fat mass 15.0 ± 6.0 kg) undergoing maintenance hemodialysis with DXA [81]. Among this cohort, 110 showed a decrease in annual fat mass, while 80 showed an increase. During the follow-up period of 5 years, 65 (34.3%) of the patients died, and Kaplan–Meier analysis demonstrated that those who lost fat mass had a significantly lower survival rate during the follow-up period compared with those who gained fat mass (*p* = 0.021), with the 5-year survival rates shown to be 58.2% and 76.3%, respectively. Furthermore, multivariate Cox regression analysis indicated that a decrease in annual fat mass (HR 0.504; 95% CI 0.264–0.961; *p* = 0.0375; loser vs. gainer) as well as the value for annual fat mass change (HR 0.855; 95% CI 0.763–0.958; *p* = 0.0072; for 1 kg increase) were significant predictors of all-cause mortality, after adjustments for age, hemodialysis duration, presence of diabetes, body mass index, serum albumin level, and other variables. An increase in annual fat mass of 1 kg was found to reduce mortality by 14.5%. These results demonstrated that a decrease in fat mass is an independent predictor for increased all-cause mortality in hemodialysis patients. Similarly, in a study of 535 adult hemodialysis patients whose body fat mass was directly measured with near-infrared interactance, 46 with body fat mass <12% showed mortality at a rate 4 times greater compared to 199 patients with body fat mass content between 24% and 36% (HR 4.01; 95% CI 1.61–9.99; *p* = 0.003), following multivariate adjustments for demographics, and surrogates of muscle mass and inflammation [82]. That study also found that in hemodialysis patients whose body fat mass was re-measured after 6 months (*n* = 411), fat loss (≤−1%) was associated with a two-times greater death risk than that of patients who gained fat (≥1%), after a multivariate adjustment (HR 2.04; 95% CI 1.05–4.05; *p* = 0.04). 

## 7. Important Role of Visceral Fat in Development of Various Clinical Outcomes as Compared to Subcutaneous Fat

### 7.1. Visceral Fat and Inflammation

Fat mass can be classified into visceral and subcutaneous based on distribution, and those are metabolically different. The metabolically more active visceral fat is a key factor in the development of insulin resistance, type 2 diabetes, hypertension, dyslipidemia, and atherosclerosis [83]. Moreover, visceral fat, which produces more inflammatory cytokines, such as interleukin 6 (IL-6) and tumor necrosis factor α (TNF-α), is characterized by inflammation and considered to be a risk factor for CVD and increased mortality [84]. In contrast, subcutaneous fat has not been consistently linked to these adverse phenomena and may reflect overall nutritional status as energy storage in both the general population and CKD patients [85]. 

Waist circumference, an indicator of visceral fat, has been shown to be associated with inflammatory markers in general population cohorts. Schrager et al. reported that waist circumference was associated with higher levels of inflammation markers than overall obesity in community-dwelling older people (age ≥65 years, 378 males and 493 females) [86]. In an evaluation of the association between adiposity and inflammation markers in 179 older adults (age 77 ± 4 years, 70% females), Brinkley et al. found that large waist circumference (≥102 cm for males, ≥88 cm for females) was more strongly correlated with inflammation markers than total fat mass determined by DXA [87].

Previous studies have shown that greater waist circumference is associated with higher levels of inflammation in hemodialysis patients [88], similar to the general population, and thus, visceral fat may also play an inflammatory role in these patients. Delgado et al. enrolled 609 patients undergoing hemodialysis (age 56.1 ± 14.3 years, 57% males, 43% diabetics) and found that waist circumference was positively correlated with CRP and IL-6 concentrations, and inversely with serum albumin and prealbumin concentration. In contrast, the total percentage of fat adjusted for waist circumference, used as a proxy for subcutaneous fat, was inversely correlated with CRP and IL-6 concentration and positively with prealbumin and albumin concentration [89]. In a study of 15,314 CKD patients who participated in the Third National Health and Nutrition Examination Survey (NHANES III), Beddhu et al. noted that abdominal obesity was associated with inflammation (CRP >3 mg/L) [90]. 

To examine the relationship between fat mass distribution and inflammation, we investigated the association of body composition, determined by DXA, and CRP in 452 hemodialysis patients (age 64 ± 11 years, hemodialysis duration 89 ± 77 months, 63% males, 37% diabetics) [91]. The patients were divided into two groups according to serum high-sensitivity CRP (hsCRP) level—normal CRP (*n* = 346; hsCRP < 0.3 mg/dL, i.e., normal level) and high CRP (*n* = 106; hsCRP ≥ 0.3 mg/dL). Fat mass in the high CRP group was significantly greater, while there was no significant difference for lean mass between the groups. Additionally, truncal fat mass (surrogate for visceral fat mass) was significantly greater in the high compared to the normal CRP group, with no significant difference in nontruncal fat mass (surrogate for subcutaneous fat mass) between them. In multiple regression analysis, truncal fat mass (β = 0.227; *p* < 0.01) was significantly and independently associated with serum hsCRP level, after adjustments for age, gender, diabetes, and other confounders (R^2^ = 0.137; *p* < 0.01), whereas nontruncal fat mass was not.

In a study of 197 CKD patients (age 52 ± 1 years, 123 males) examined shortly before starting dialysis, Axelsson et al. reported that truncal fat mass, estimated by DXA, was significantly positively correlated with both CRP (ρ = 0.23; *p* < 0.01) and IL-6 (ρ = 0.21; *p* < 0.01) levels [92]. Kaysen et al. examined visceral adipose tissue using MRI in 48 patients with prevalent hemodialysis and showed that ceruloplasmin, an acute phase inflammatory protein, was strongly associated with visceral adipose tissue [93]. Further, Gohda et al. examined the visceral fat area shown in CT results in 80 patients with prevalent hemodialysis and found that the visceral fat area was a predominant determinant of CRP [94]. These results suggest that visceral or truncal fat mass is an important contributor to increased inflammation in hemodialysis patients, similar to the general population.

### 7.2. Visceral Fat and CVD Risk

Visceral fat was found to be associated with CVD risk in a general population study. Canoy et al. examined the prospective relationship between fat distribution indices and coronary heart disease among 11,117 males and 13,391 females ranging from 45 to 79 years old [95]. During a mean follow-up period of 9.1 years, 1708 males and 892 females developed coronary heart disease. The risk for developing subsequent coronary heart disease was increased with elevation of waist-to-hip ratio and waist circumference. The HR value (95% CI) of the top compared to the bottom fifth of waist-to-hip ratio was 1.55 (1.28–1.73) in males and 1.91 (1.44–2.54) in females, after adjustments for BMI and other coronary heart disease risk factors. Additionally, risk estimates for waist circumference without hip circumference adjustment were 10% to 18% lower. 

Visceral fat might also be associated with outcomes including cardiovascular events in hemodialysis patients [96]. In an Asian hemodialysis cohort (*n* = 91, age 58.7 ± 12.5 years, 56.0% males) analyzed over a three-year period, Wu et al. reported that central obesity, determined based on a waist circumference ≥90 cm in males and ≥80 cm in females, was a significant predictor of cardiovascular events (HR 4.91; 95% CI 1.3–18.9; *p* = 0.02) and all-cause hospitalization (HR 1.83; 95% CI 1.1–3.1; *p* = 0.03), shown in multivariate Cox regression analysis results [97]. 

The negative metabolic consequences of excess visceral fat are preserved in CKD patients [98]. In the study conducted by Sanches et al. of 122 patients with CKD and not yet receiving dialysis therapy (age 55.3 ± 11.3 years, 75 males and 47 females, 30% diabetics, BMI 27.1 ± 5.2 kg/m^2^, eGFR 35.4 ± 15.2 mL/min/1.73 m^2^), waist circumference was strongly correlated with visceral fat determined by CT (r = 0.75 for males, r = 0.81 for males, *p* < 0.01), and visceral fat was associated with risk factors for cardiovascular disease, such as triacylglycerol levels [99]. In another investigation of 1669 subjects with CKD (age 70.3 years, 56% females), defined as a baseline eGFR of 15–60 mL/min/1.73 m^2^, Elsayed et al. examined the association between waist-to-hip ratio and risk for cardiac events (myocardial infarction, fatal coronary disease) [100]. During a mean follow-up period of 9.3 years, there were 334 cardiac events. Using multivariable-adjusted Cox models, the highest waist-to-hip ratio group (*n* = 386) was found to be associated with increased risk of cardiac events as compared with the lowest waist-to-hip ratio group (*n* = 590, HR 1.36; 95% CI 1.01–1.83). 

### 7.3. Subcutaneous Fat and Metabolic Risk

In contrast to visceral fat, subcutaneous fat may have beneficial metabolic effects and possibly reflects overall nutritional status as energy storage in the general population [101]. In 115 healthy, overweight/moderately obese adults with BMI ranging from 25 to 36.9 kg/m^2^, McLaughlin et al. found that despite nearly identical mean BMI values in the insulin-resistant and insulin-sensitive groups, visceral adipose tissue, quantified by CT results, was significantly higher in the insulin resistance group, whereas subcutaneous adipose tissue was significantly lower [102]. Inclusion of both visceral adipose tissue and subcutaneous adipose tissue in the same multiple logistic regression analysis demonstrated independent associations, in opposite directions, for both visceral (OR: 1.77; 95% CI 1.04–3.02) and subcutaneous (OR: 0.56; 95% CI 0.34–0.94) adipose tissue with insulin resistance as compared to insulin sensitivity, after adjusting for BMI and gender.

Tanko et al. enrolled 1356 elderly females from 60 to 85 years old and reported that subcutaneous fat, determined by DXA, had an independent negative correlation with both atherogenic metabolic risk factors, such as glucose and lipid metabolites, and aortic calcification, assessed by lateral radiograph findings, in contrast to visceral fat, used as a central adiposity surrogate [103]. The most severe instances of insulin resistance—dyslipidemic syndrome and aortic calcification—were found in subjects with high central and low subcutaneous fat percentages. In a study of 3001 participants in the Framingham Heart Study who were free from clinical cardiovascular disease (mean age 50 years, 48% females), Fox et al. found that visceral adipose tissue but not subcutaneous abdominal adipose tissue, both determined by CT results, contributed significantly to metabolic risk factors, after adjustments for covariant factors, including BMI [85].

The effects of subcutaneous and visceral fat on metabolic risk and inflammation have also been observed in animal models. In mice given a high-fat diet, glucose tolerance was improved in those implanted intra-abdominally with subcutaneous adipose tissue as compared to mice with epididymal visceral adipose tissue implanted intra-abdominally [104]. Mice that received subcutaneous adipose tissue also displayed a marked reduction in the plasma concentration of several proinflammatory cytokines, such as TNF-α and IL-17.

### 7.4. Visceral Fat and Mortality

Several studies that included general population subjects and CKD patients [105] have shown that increased visceral fat is associated with a higher risk of mortality. Leitzmann et al. prospectively examined waist circumference in relation to cause-specific death in a large cohort of males and females in the United States (*n* = 225,712). Increased waist circumference was consistently associated with increased risk of death due to any cause as well as major causes of death, including CVD, independent of BMI, age, gender, ethnicity, smoking status, and alcohol intake [106]. Similarly, Lahmann et al. reported that a higher waist-to-hip ratio was a strong predictor of all-cause mortality independent of percentage of body fat mass in 16,814 middle-aged and older females examined in Sweden [107].

Postorino et al. performed a prospective cohort study of 537 hemodialysis patients (age 63 ± 15 years) [32]. Using BMI-adjusted Cox models, waist circumference was found to be a direct predictor of all-cause and cardiovascular mortality (*p* < 0.001), whereas BMI showed an inverse relationship (*p* < 0.001) with those outcomes. The rates of overall and cardiovascular death were maximum in patients with a relatively lower BMI score (below median) and higher waist circumferences (at least median), but minimal in patients with a higher BMI score (at least median) and small waist circumferences (below median). The prognostic power of waist circumference per 10 cm increase for all-cause (HR 1.23; 95% CI 1.02–1.47; *p* = 0.03) and cardiovascular (HR 1.37; 95% CI 1.09–1.73; *p* = 0.006) mortality remained significant after adjustments for cardiovascular comorbidities, as well as traditional and emerging risk factors. Waist-to-hip ratio was also demonstrated to be related to all-cause (*p* = 0.009) and cardiovascular (*p* = 0.07) mortality. Similarly, higher BMI has been found to be protective against and greater waist circumference predictive of mortality in an elderly population [108], as well as kidney transplant patients [109].

In a study of 97 hemodialysis patients, Xiong et al. found that visceral fat determined by BIA was associated with cardiovascular events (HR 9.21; 95% CI 1.49–56.76; visceral fat area ≥71.3 cm^2^ vs. <71.3 cm^2^; *p* = 0.017), cardiovascular mortality (HR 1.11; 95% CI 1.01–1.22; 1-cm^2^ increase in fat mass area; *p* = 0.035), and all-cause mortality (HR 1.08; 95% CI 1.02–1.14; 1-cm^2^ increase in fat mass area; *p* = 0.011) [110]. Okamoto et al. followed 126 patients on maintenance hemodialysis for 60 months and reported multivariate Cox proportional hazards analysis results showing that a visceral fat area of >71.5 cm^2^, determined by CT, was an independent predictor of cardiovascular death (HR 4.46; 95% CI 1.24–16.05; *p* = 0.022) [111].

In a study of 5805 CKD Stage 1–4 patients with BMI ≥ 18.5 kg/m^2^, Kramer et al. showed that the association between waist circumference and all-cause mortality was fairly linear, and HR values for mortality were significantly higher for waist circumference ≥98 cm in females and ≥112 cm in males, as compared to the reference waist circumference values (<80 and <94 cm, respectively) [112]. After adjustments for all covariates, including BMI, HR for all-cause mortality for waist circumference ≥108 cm in females and ≥122 cm in males was 2.09 (95% CI 1.26–3.46) as compared to the reference values. After fully adjusting for continuous variables, each 1 cm increase in waist circumference was associated with a 2% increase in risk of mortality (95% CI 1.01–1.04).

### 7.5. Subcutaneous Fat and Mortality

Visceral and subcutaneous adipose tissues have different metabolic and inflammatory characteristics, and they thus may have an opposing influence on several outcomes, including mortality. In a general population study, preferential fat deposition in subcutaneous and visceral locations was suggested to be more important than the total amount of body fat in regard to mortality. Lee et al. investigated the relationship between body fat distribution and all-cause mortality in 32,593 subjects who underwent an abdominal CT examination as part of a health check-up [113]. There were 253 deaths during the mean follow-up period of 5.7 years. Their findings showed that an increased visceral fat area was related to increased all-cause mortality, while an increased subcutaneous fat area was associated with a decrease in all-cause mortality. However, in multivariate Cox proportional hazard regression analysis, only the visceral fat area was found to be independently associated with all-cause mortality. 

The association between subcutaneous fat and mortality has also been observed in regard to other conditions, such as cancer [114] and cirrhosis [115]. In a study conducted by Ebadi et al., the association between subcutaneous fat and mortality in 1473 gastrointestinal and respiratory cancer and 273 metastatic renal cell carcinoma patients was investigated using CT results. A low subcutaneous adipose tissue index (<50.0 cm^2^/m^2^ in males, <42.0 cm^2^/m^2^ in females) was shown to be independently associated with increased mortality (HR 1.26; 95% CI 1.11–1.43; *p* < 0.001) and shorter survival (13.1 months; 95% CI 11.4–14.7) compared to patients with a high subcutaneous adipose tissue index (19.3 months; 95% CI 17.6–21.0; *p* < 0.001) [116]. Antoun et al. also reported findings of 120 patients with metastatic castration-resistant prostate cancer, which showed that those with a higher subcutaneous adipose tissue index had significantly longer overall survival [117]. The median survival was 15 months (95% CI 9–18) for patients with a subcutaneous adipose tissue index lower than the median value and 18 months (95% CI 13–30) for those with a subcutaneous adipose tissue index above that (*p* = 0.008). 

The relationships of fat and muscle mass with mortality were examined by Huang et al. in 1709 hemodialysis patients (age 57.7 ± 14.0 years, hemodialysis duration 3.7 ± 4.4 years, 56% females, 44% diabetics) who participated in the hemodialysis study (HEMO) [118]. During a median follow-up period of 2.5 years, there were 802 deaths. In adjusted models with continuous covariates, higher triceps skinfold thickness was significantly associated with decreased risk of mortality, while higher mid-arm muscle circumference showed a trend toward decreased mortality. The HR values per 1 S.D. increase were 0.84 (95% CI 0.76–0.92) for triceps skinfold thickness and 0.93 (95% CI 0.86–1.00) for mid-arm muscle circumference. The highest quartiles of triceps skinfold thickness and mid-arm muscle circumference were significantly associated with lower all-cause mortality in comparison with the lowest quartile in adjusted models. Furthermore, triceps skinfold thickness and mid-arm muscle circumference were independently associated with lower risk of mortality when both variables were combined in the same multivariable model. Considering that skinfold thickness is a measure of subcutaneous fat, their results indicated that a high level of subcutaneous fat mass may be associated with better survival in hemodialysis patients. Subcutaneous fat mass has a far greater contribution to total fat mass, as visceral fat mass accounts for only 7–15% of total body fat mass, and thus, it may substantially influence the association of total fat mass and outcomes. 

## 8. The Mechanism by Which Maintained Adiposity Improves Survival in Hemodialysis Patients

In addition to its advantage as a source of fuel, adipose tissue can also exert its beneficial effects through multiple mechanisms, both directly and indirectly in hemodialysis patients [44]. In addition to the direct effect of adipocytes in maintaining good health, adipose tissue produces the TNF-α-soluble receptor that attenuates the adverse effects of TNF-α itself [119], and obese individuals have higher lipoprotein concentrations, which counteract the inflammatory effects of circulating endotoxins.

Subcutaneous fat is positively associated with insulin sensitivity [102] and a slower rate of lipolysis and free fatty acid release into the circulation. Several studies have suggested that subcutaneous fat may exert protective effects against inflammation [91,104]. Subcutaneous fat is considered to be the main source of adiponectin, which is involved in a variety of physiological functions, including energy regulation, inflammation, and insulin sensitivity [120].

Furthermore, overweight and obese individuals have a higher absolute amount of muscle mass thanks to an excess load of increased adiposity. This increased amount of lean tissue might confer an additional protective edge during times of catabolism [121].

Conversely, the absence of adipose tissue causes metabolic dysfunction, including insulin resistance, hyperglycemia, hyperlipidemia, and fatty liver, which can be completely reversed with the transplantation of adipose tissue [122]. Similarly, reductions in total body fat are associated with decreased humoral immunity [123].

## 9. Conclusions

Nutritional status is closely associated with outcomes including mortality in hemodialysis patients and shows continual fluctuations; thus, regular examinations for assessment of nutrition factors are necessary. Although obesity and excessive fat mass are linked to CVD and mortality in the general population, fat mass is considered to be an important nutritional indicator in hemodialysis patients, who are typically malnourished; thus, its measurement is important for a good understanding of nutritional status. It is also important to note that fat mass is classified into visceral and subcutaneous fat based on distribution, and that those have metabolic differences. Visceral fat is closely associated with metabolism abnormalities and inflammation and considered to be a risk factor for adverse outcomes, such as CVD and mortality, in the general population as well as in hemodialysis patients. In contrast, subcutaneous fat may be protective against wasting and catabolism in patients undergoing hemodialysis. However, fat mass may have potentially beneficial effects on important outcomes in hemodialysis patients, as accumulating evidence suggests that fat mass distribution (i.e., visceral fat and subcutaneous fat) plays a more important role in these beneficial effects.
